# Cancer apelin receptor suppresses vascular mimicry in malignant melanoma

**DOI:** 10.3389/pore.2023.1610867

**Published:** 2023-01-27

**Authors:** Koichi Inukai, Kazuyoshi Kise, Yumiko Hayashi, Weizhen Jia, Fumitaka Muramatsu, Naoki Okamoto, Hirotaka Konishi, Keigo Akuta, Hiroyasu Kidoya, Nobuyuki Takakura

**Affiliations:** ^1^ Department of Signal Transduction, Research Institute for Microbial Diseases, Osaka University, Suita, Japan; ^2^ Department of Integrative Vascular Biology, Faculty of Medical Sciences, University of Fukui, Fukui, Japan; ^3^ World Premier Institute Immunology Frontier Research Center, Integrated Frontier Research for Medical Science Division, Osaka University, Suita, Japan; ^4^ Institute for Open and Transdisciplinary Research Initiatives (OTRI), Osaka University, Suita, Japan; ^5^ Center for Infectious Disease Education and Research, Osaka University, Suita, Japan

**Keywords:** epithelial-mesenchymal transition, melanoma, apelin, apelin receptor, neovascularization

## Abstract

Several reports indicate that apelin is often over-expressed in tumors, and therefore it has been suggested that the apelin–apelin receptor (APJ) system may induce tumor progression. In contrast, our previous research revealed high expression of the apelin–APJ system in tumor blood vessels, suggesting its involvement in the regulation of tumor vessel formation and normalization, resulting in the suppression of tumor growth by promoting the infiltration of T cells. Thus, the effect of the apelin–APJ system on tumors remains controversial. In this report, to clarify the effect of apelin in tumor cells, we analyzed the function of APJ in tumor cells using APJ knock out (KO) mice. In APJ-KO mice, Apelin overexpression in B16/BL6 (B16) melanoma cells induced greater tumor growth than controls. In an APJ-KO melanoma inoculation model, although angiogenesis is suppressed compared to wild type, no difference is evident in tumor growth. We found that APJ deficiency promoted vascular mimicry in tumors. *In vitro*, cultured APJ-KO B16 cells demonstrated a spindle-like shape. This phenotypic change was thought to be induced by epithelial–mesenchymal transition (EMT) based on evidence that APJ-KO B16 cells show persistently high levels of the mesenchymal maker, Zeb1; however, we found that EMT did not correlate with the transforming growth factor-β/smad signaling pathway in our model. We propose that apelin-APJ system in cancer cells induces tumor growth but negatively regulates EMT and tumor malignancy.

## Introduction

Apelin is a bioactive oligopeptide identified as a ligand for the G-protein coupled receptor (GPCR), APJ. Apelin is expressed in various tissues and is involved in a variety of physiological functions, from organogenesis to homeostasis. In addition to apelin, a new ligand for APJ, apelin early ligand A (APELA), also known as Elabela (ELA) or Toddler, has been identified and was shown to be important in angiogenesis and embryonic development [1,2,3]. Moreover, gene and protein expression of apelin and APJ are modulated by fluid flow depending on the magnitude of shear stress [4], and it is possible that APJ activation is induced by such mechanical stress.

We previously described the function of the apelin–APJ system in physiological and pathological vascular development [5, 6]. In a physiological setting, we found that apelin is involved in regulating the caliber size of blood vessels, inducing the formation of larger caliber-sized capillaries by exerting cell–cell aggregation of endothelial cells (ECs). Moreover, we found that apelin suppressed vascular permeability by stabilizing the membrane expression of vascular endothelial (VE)-cadherin [7]. Furthermore, we identified the function of apelin/APJ in the juxtapositional alignment of arteries and veins by mainly regulating the movement of veins to arteries [8].

Recently, apart from cardiovascular development, high expression of apelin and APJ has also been observed in tumor tissue compared to normal tissue, suggesting their involvement in the regulation of tumor vessel formation and normalization [9,10,11]. We also found that apelin was involved in the formation of a vascular system in tumors, showing a variety of effects. For example, Colon 26 tumors showing apelin overexpression in mice after subcutaneous implantation inhibited tumor growth. Tumor growth by LLC or MC38 cells was enhanced significantly in apelin-knockout (KO) mice [12, 13]. However, in other reports, apelin induces the growth of tumors by enhancing the tumor vasculature [14, 15]. These findings suggested that apelin might have diverse functions in tumor formation depending on the tumor microenvironment.

In our previous study, APJ activation by apelin induced the morphological and functional maturation of blood vessels in tumors, resulting in the suppression of tumor growth by promoting the infiltration of T cells into the central region of the tumor and thereby robustly inducing apoptosis in tumor cells [12, 13]. However, several lines of evidence indicate that apelin is often over-expressed in tumors, suggesting that this factor may induce tumor growth because its expression correlates with tumor malignancy [10].

Discrepancies in tumor growth affected by apelin may due to the expression of its receptor, APJ, in the tumor microenvironment. Usually, APJ is expressed on vascular ECs during embryogenesis and is weakly expressed in ECs after birth. When angiogenesis is induced, ECs may re-express APJ; however, tumor cells themselves are suggested to express APJ with a wide range of expression. This may affect complicity in the tumor growth affected by apelin. To elucidate the effect of apelin on tumor cells, the elimination of APJ expression in host cells (mainly ECs) is required. In the present study, therefore, we analyzed the function of APJ in tumor cells by using APJ-KO mice and observed the role of APJ in cancer cells.

## Methods

### Mice and cell lines

Wild-type C57BL/6 mice were purchased from Japan SLC (Shizuoka, Japan). APJ-KO mice were gifts from Prof. Fukamizu [16]. All mice were used between 8 and 11 weeks of age. Animals were housed in environmentally-controlled rooms in an animal experimentation facility at Osaka University. All mouse experiments were performed under the guidelines of the Osaka University Committee for Animal and Recombinant DNA Experiments (Approval number 4784).

B16/BL6, a murine melanoma cell line, was purchased from the Riken Cell Bank (Tsukuba, Japan) and maintained in DMEM (Sigma–Aldrich, St. Louis, MO, United States) supplemented with 10% fetal bovine serum (FBS, Sigma–Aldrich) and 1% penicillin/streptomycin (Life Technologies, Tokyo, Japan). B16/BL6 cells were starved and then stimulated with LY364947 (10 μM; Selleck Biotech, Huston, TX, United States) or dimethyl sulfoxide (Nacalai Tesque, Inc., Kyoto, Japan) for 24 h. Methods for the generation of apelin-overexpressing cancer cells were previously described [12].

### CRISPR-Cas9 method for APJ-KO tumor cells

To establish APJ-KO cells using a CRISPR/Cas9 genome editing system, single-guide RNAs were designed using a CRISPRdirect Web application (https://crispr.dbcls.jp) for the mAPLNR first exon using pSpCas9(BB)-2A-Puro (PX459) V2.0 (plasmid number 62988; Addgene, Watertown, MA, United States). The two-pair gene-specific single-guide RNA sequences were 5′-CAC CGA GGC CAG TCA AAC TCC CGG T - 3′(sense), 5′- AAA CAC CGG GAG TTT GAC TGG CCT C -3′ (antisense), 5′-CAC CGA GGT GCA GGC TTG GCG AAA T′ (sense), and 5′- AAA CAT TTC GCC AAG CCT GCA CCT C-3′ (antisense). The oligos were ligated into a BbsI site of PX459. The constructed targeting vector was transfected into a B16/BL6 cell line. Clones were selected, and elimination of APJ expression at mRNA and protein levels was detected through real-time quantitative PCR (RT–qPCR) and western blot analysis, respectively (as described below).

### *In vivo* tumor subcutaneous inoculation

B16/BL6 cells (1 × 10^7^ cells/mL per mouse in 150 µL PBS) were inoculated subcutaneously into WT C57BL/6 or APJ-KO mice (8–11 weeks of age). Tumors were dissected 14 days after implantation. Tumor volumes were measured with calipers every 5–7 days and calculated as follows: width × width × length × 0.5.

### Western blot analysis

In APJ western blot analysis, cell extracts were prepared with GPCR extraction and stabilization reagent (Thermo Fisher Scientific, Waltham, MA, United States) for APJ or with radioimmunoprecipitation assay buffer (Thermo Fisher Scientific) according to the manufacturer’s protocol. Proteins were separated by sodium dodecyl sulfate–polyacrylamide gel electrophoresis, transferred to polyvinylidene difluoride membranes, and blocked with 5% skimmed milk in 0.1% Tris-buffered saline and Tween 20. Membranes were subsequently incubated with antibodies against APJ (5H5L9; Thermo Fisher Scientific), p-Smad2, Smad2, p-Smad3, Smad3 (Cell Signaling Technology, Danvers, MA, United States), or glyceraldehyde-3-phosphate dehydrogenase (Millipore, Temecula, CA, United States), and then developed with horseradish peroxidase–conjugated secondary antibodies (Jackson ImmunoResearch Laboratories, West Grove, PA, United States). Antibody binding was visualized using electrochemiluminescence reagents (GE Healthcare, Milwaukee, WI, United States).

### Transwell assay

Cell migration was evaluated using a transwell assay. For the analysis of cell migration, cells were suspended in DMEM at a density of 1 × 10^6^ cells/mL and added to the upper chamber (200 μL/chamber); 750 μL of medium (DMEM containing 10% FBS) was added to the lower chamber. After 14 h, cells on the top membrane surface of the upper chamber were removed using a cotton swab, and the infiltrating cells on the bottom surface were fixed in 4% paraformaldehyde for 1 min, stained with hematoxylin and eosin, and visualized and counted using a microscope.

### Tube formation assay

Each well of a 96-well plate was coated with 60 μL of growth factor–reduced UltiMatrix (R&D Systems, Inc., Minneapolis, MN, United States) for 30–60 min at 37°C. Melanoma cells were seeded onto the gel surface at a density of 4.0 × 10^4^ cells per well in a volume of 200 μL medium. Network formation was analyzed after a 15-h incubation. Images were analyzed with ImageJ and Angiogenesis analyzer software [17].

### Immunostaining

Tumor sections were rehydrated in PBS and blocked with normal goat serum in PBS for 20 min at room temperature. Primary antibodies used were: rabbit anti-APJ (5H5L9; Thermo Fisher Scientific; 1:200), hamster anti-CD31 (Millipore; 1:200), and rat anti-GFP (BioLegend, San Diego, CA, United States; 1:200) with overnight incubation. Following three washes with PBS, sections were incubated with Alexa Fluor-conjugated secondary antibody (Thermo Fisher Scientific) and diluted 1:200 in blocking solution for 60 min. After three washing steps with PBS, sections were mounted using Vectashield (Vector Laboratories, Burlingame, CA, United States) under coverslips. Stained sections were examined using confocal microscopy (TCS/SP8 or TCS/SP5; Leica Microsystems, Wetzlar, Germany).

### Angiogenesis analysis

To quantify the length and density of blood vessels, CD31-positive immunofluorescence images were analyzed with the computational analysis tool, Angiotool (version 0.6; National Institutes of Health, National Cancer Institute, Bethesda, MD, United States) [18]. It performs the analysis of vascular morphometric parameters like vessel length and density. Tumor sections were scanned under low-power magnification (×200) to identify areas of highest CD31-positive vessel density. All images shown are representative of more than five independent experiments.

### qRT–PCR analysis

Briefly, total RNA was extracted from cells using RNeasy-plus mini kits (Qiagen, Hilden, Germany) and reverse-transcribed using a PrimeScript RT reagent kit (Takara, Shiga, Japan) according to the manufacturer’s protocol. Quantitative real-time PCR analysis was performed with TB Green Premix Ex Taq II (Takara) using a LightCycler 96 System (Roche Diagnostics GmbH, Penzberg, Germany). Primers are described in Supplementary Table S1. Results were normalized to GAPDH using a comparative threshold cycle method.

## Results

### APJ is expressed in malignant melanoma cells

**FIGURE 1:**
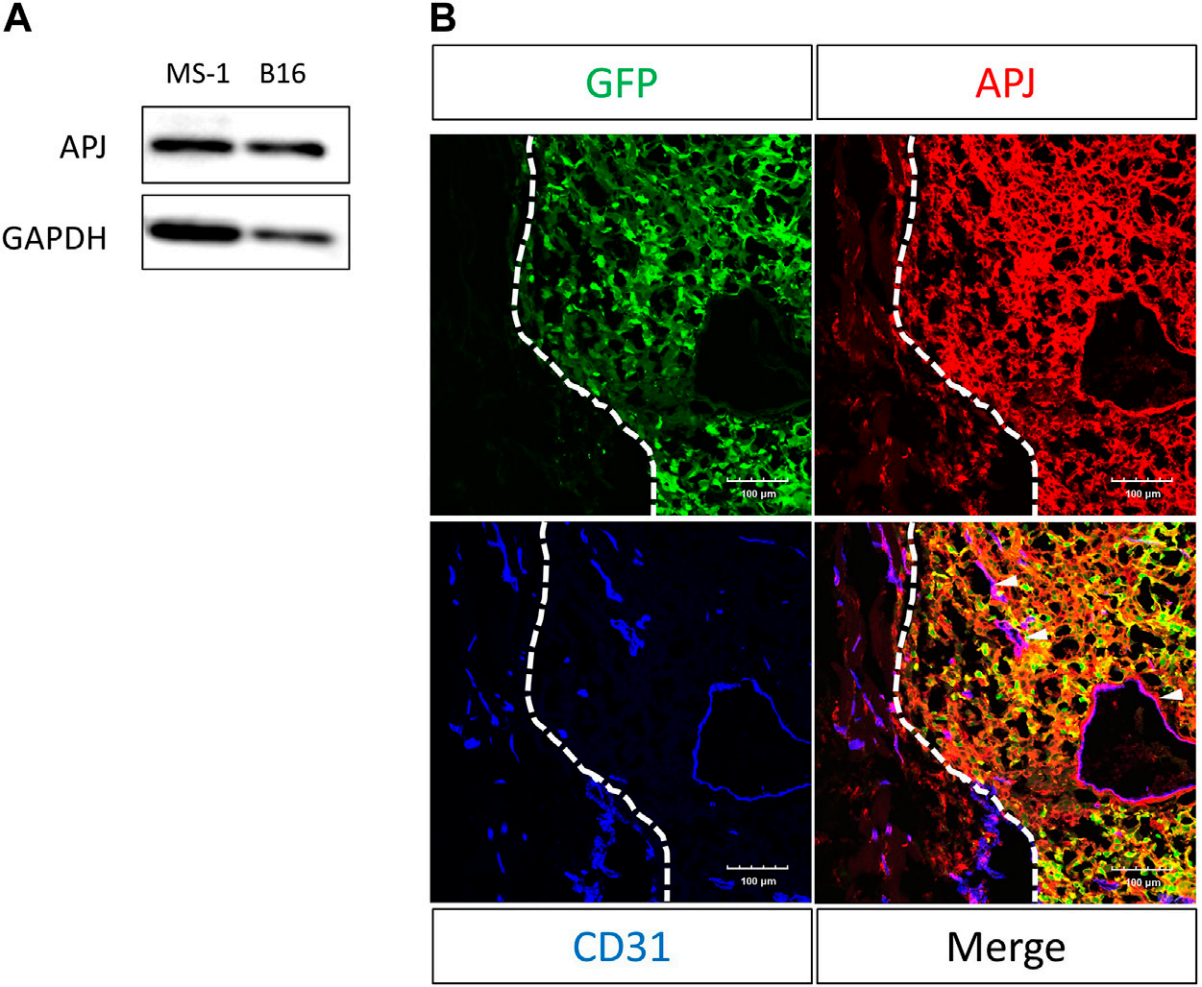
APJ is expressed in B16/BL6 malignant melanoma cells. **(A)** Western blot analysis with anti-APJ and anti–glyceraldehyde-3-phosphate dehydrogenase (GAPDH) antibodies in whole cell lysates, as indicated. **(B)** Representative image of a B16/BL6 tumor from a mouse (*n* = 4). Tumor sections were stained with anti-green fluorescent protein (GFP) (green), -APJ (red), and -CD31 (blue) antibodies. Dashed lines indicate the tumor edge. White arrowheads indicate APJ-positive blood vessels. Scale bars = 100 μm.

We examined melanoma cells for the expression of APJ protein. We found that B16/BL6 (B16) melanoma cells strongly expressed this compared with murine ECs (MS-1), which were previously reported to express APJ (Figure 1A). As observed in cultured cells, immunohistochemical staining in tumor tissues of C57/BL6 wild-type mice subcutaneously inoculated with B16 cells overexpressing green fluorescent protein (GFP) revealed that B16 melanoma cells (GFP positive) expressed APJ protein (Figure 1B); APJ expression was equivalently observed in tumor ECs.

### APJ expressed in B16 melanoma cells induces tumor growth by apelin

**FIGURE 2:**
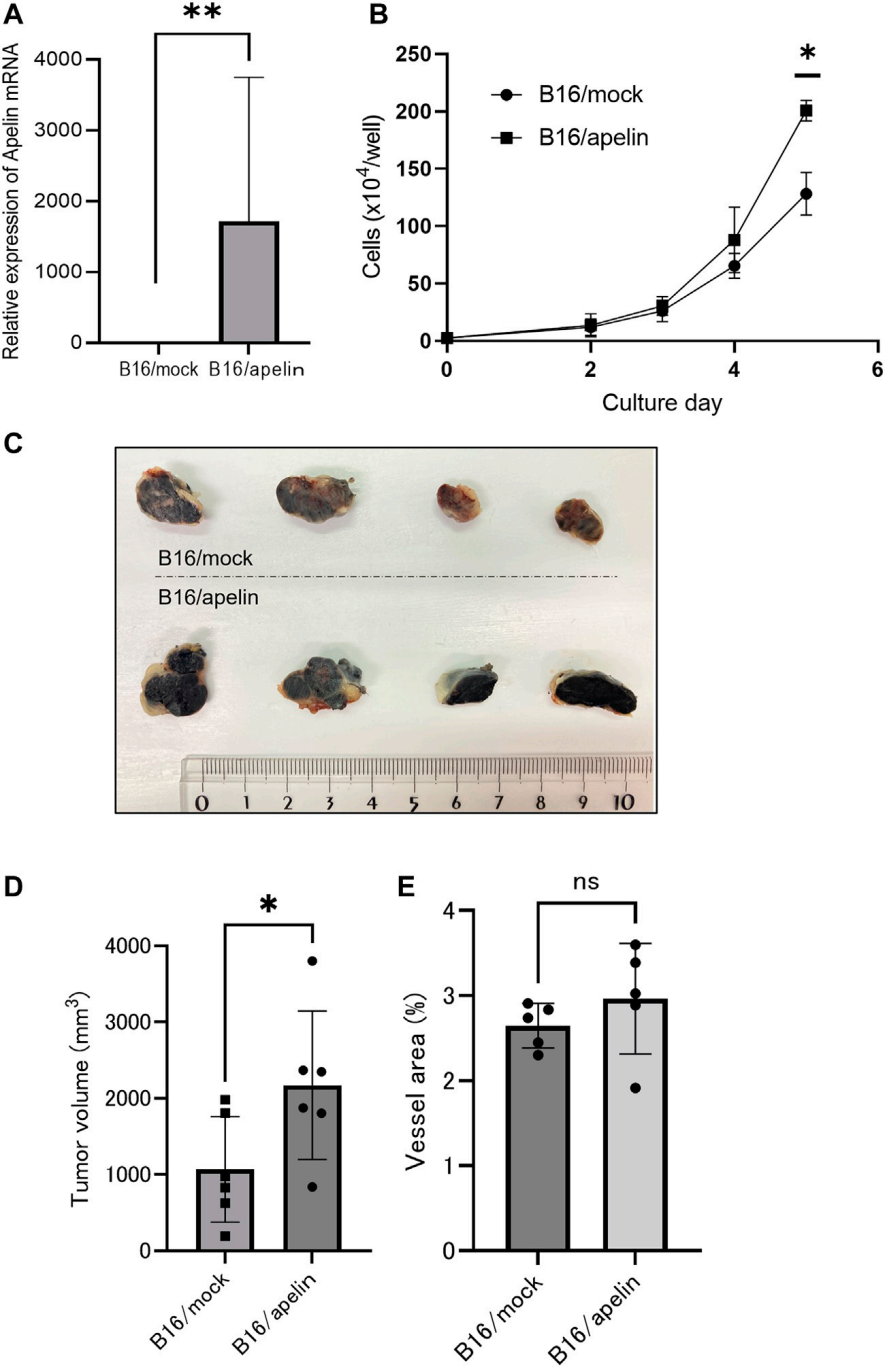
APJ induces cell proliferation and tumor growth by apelin. **(A)** Quantitative evaluation of apelin mRNA expression in B16 cells transfected with an apelin expression plasmid (*n* = 3). **(B)** *In vitro* growth of apelin-overexpressing B16 (B16/apelin) and control B16 (B16/mock-transfected) cells (*n* = 3). **(C, D)** Comparison of tumor growth by control B16/mock-transfected cells with B16/apelin cells. **(C)** Representative gross appearance (*n* = 4). **(D)** Quantification of tumor sizes (*n* = 6). **(E)** Quantification of blood vessel areas in B16/mock-transfected and B16/apelin tumors on day 14 after B16 cell inoculation of APJ-knockout (KO) mice (*n* = 6). Data are expressed as mean ± SD, *p < 0.05, **p < 0.01, ns: not significant.

To analyze the effect of apelin on APJ in cancer cells, we overexpressed apelin in B16 cells (B16/apelin; Figure 2A; *p* < 0.01). When cell proliferation was compared in mock-transfected B16 cells and those transfected with apelin, we found that apelin significantly promoted the proliferation of apelin-overexpressing B16 cancer cells (Figure 2B; *p* < 0.05). Next, to evaluate whether or not cell proliferation by apelin reflects *in vivo* tumor growth, we transplanted cancer cells into APJ-KO mice to eliminate the effect of apelin on cells other than cancer cells, especially ECs. We subcutaneously inoculated B16/apelin or mock-transfected control cells into APJ-KO mice and found that B16/apelin mice showed significantly higher tumor growth than B16/mock-transfected control mice (Figures 2C, D; *p* < 0.05). In this tumor model, the density of blood vessels in B16/apelin tumors did not significantly differ from that in B16/mock-transfected control tumors (Figure 2E). Taken together, these data suggested that APJ deficiency in the tumor host microenvironment does not affect tumor angiogenesis when B16 cells were used as tumor models. An excess amount of apelin induced the growth of B16 melanoma cells by affecting APJ in tumor cells.

### Lack of APJ in B16 melanoma cells attenuates angiogenesis but does not affect tumor growth

**FIGURE 3:**
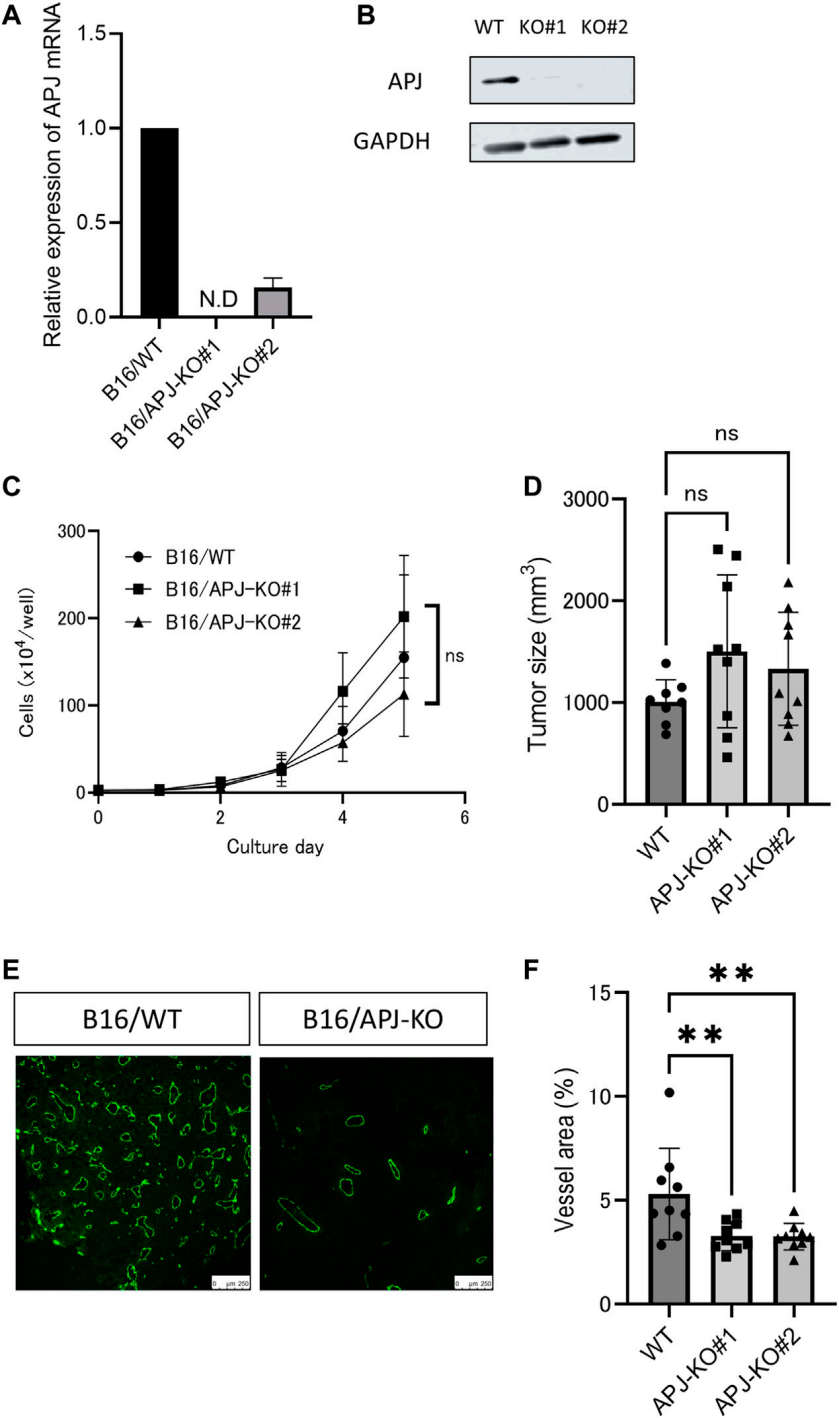
APJ knockout in melanoma retains tumor growth without angiogenesis. **(A)** Real-time quantitative PCR of APJ mRNA expression in B16/wild-type (WT) and APJ knock-out (KO) B16 clones (#1, 2) (*n* = 3). **(B)** Western blot analysis of cell lysates from B16/WT, B16/APJ-KO clones. Glyceraldehyde-3-phosphate dehydrogenase (GAPDH) protein was used as a loading control. **(C)** *In vitro* growth of B16/WT and B16/APJ-KO cells (*n* = 3). **(D)** The sizes of tumors derived from B16/WT and B16/APJ cell lines subcutaneously inoculated into WT mice on day 14 (*n* = 8/WT vs. *n* = 9/APJ-KO). **(E)** Representative immunofluorescence staining for CD31 in B16/WT and B16/APJ-KO tumors (*n* = 8/WT vs. *n* = 9/APJ-KO). Scale bars = 250 μm. **(F)** Quantitative evaluation of vessel areas in tumors derived from B16/WT and B16/APJ-KO (#1, 2) cell lines (*n* = 8/WT vs. n = 9/APJ-KO). Data are expressed as mean ± SD, KO, knockout; N.D, not detected; WT, wild type; **p < 0.01; ns, not significant.

We found that overexpression of apelin in B16 melanoma cells induced the growth of tumors. Therefore, we next observed whether or not a lack of APJ in B16 cells would affect tumor growth. To assess this, we knocked out the *APJ* gene in B16 cells by a clustered regularly interspaced short palindromic repeats/CRISPR-associated protein (CRISPR/Cas9) method and established two sublines (B16/APJ-KO#1, 2). The mRNA (Figure 3A) and protein (Figure 3B) levels of APJ in cell lines were analyzed. When cells were cultured, we found no significant difference in cell growth between wild-type (WT) and APJ-KO B16 cell lines (Figure 3C).

Next, these cancer cell lines were subcutaneously inoculated into C57/BL6 WT mice and the growth of tumors was evaluated. All three tumors (B16/WT, B16/APJ-KO#1, 2) showed similar tumor growth. We calculated the tumor volume on day 14 after the inoculation of tumor cells. Unexpectedly, we found that the elimination of APJ from B16 cells did not affect the growth of tumors (Figure 3D). Tumor vascular development is a major factor in regulating tumor growth in the tumor microenvironment and therefore we next evaluated the vascular density of each tumor. Initially, we suspected that APJ deficiency induced tumor angiogenesis and compensated tumor proliferation that should be observed in the presence of an apelin/APJ system on B16 cells. However, unexpectedly, tumors generated by B16/WT cells showed a higher vascular density than tumors by B16/APJ-KO cells (Figures 3E, F; *p* < 0.01). This suggested that progression of tumor growth by B16/APJ-KO is induced by factors other than angiogenesis factors.

### APJ deficiency in B16 cells promotes vascular mimicry

**FIGURE 4:**
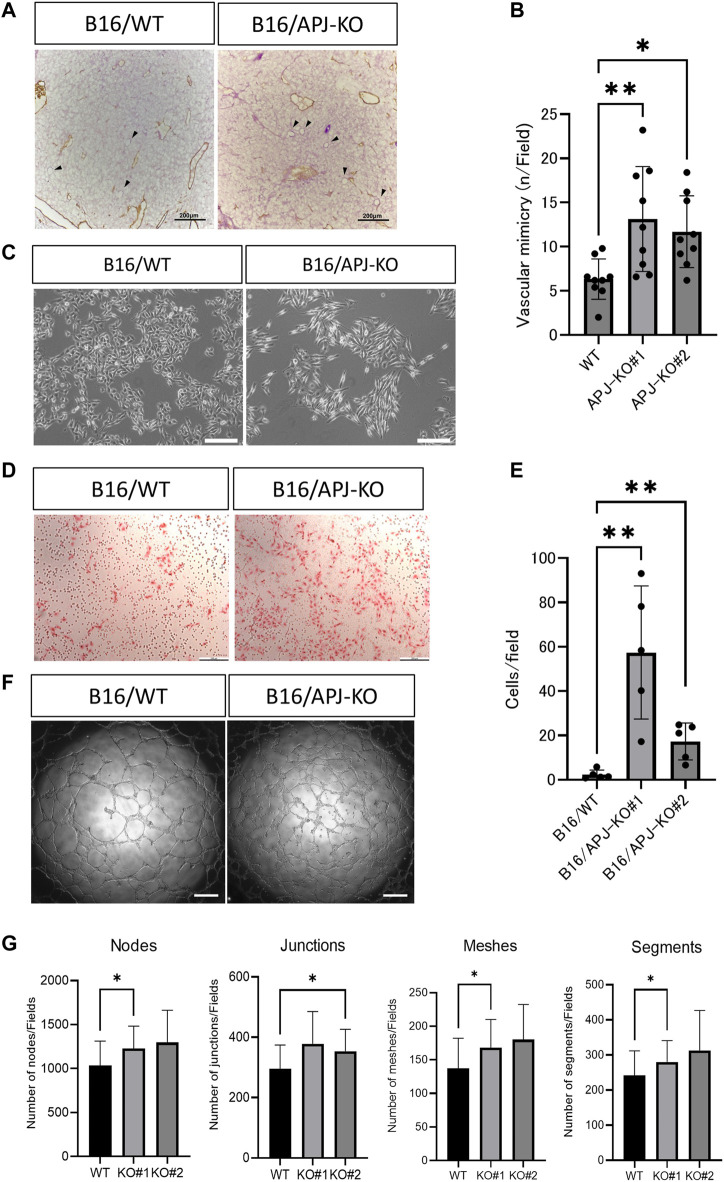
APJ knock out in B16 cells promotes vascular mimicry *in vivo* and *in vitro.* **(A)** Representative microscopic images of B16/wild-type (WT) and B16/APJ-knockout (KO) cells with double staining of CD31 (DAB; brown) and PAS (pink). Arrowheads indicates vessel-like structures induced by vascular mimicry (CD31 negative and PAS positive). Scale bars = 200 μm. **(B)** Quantitative evaluation of the vessel number induced by vascular mimicry in B16/WT and B16/APJ-KO (#1, 2) (*n* = 9) cells. **(C)** Phase contrast light microscopic images of cultured cells. Scale bars = 500 μm. **(D)** Microscopic images of cells that migrated through the transwell membrane in the migration assay (hematoxylin and eosin stain, scale bars = 200 μm. (E) The quantitation of cells that migrated through the transwell membrane in the migration assay (*n* = 5). **(F)** Microscopic images of a tube formation assay (B16/WT vs. B16/APJ-KO). Scale bars = 500 μm. **(G)** Quantitative analysis of several parameters of tube-like structures observed in a tube formation assay using ImageJ software (*n* = 9). Data are expressed as mean ± SD, *p < 0.05, **p < 0.01.

In order to maintain the same volume between B16/WT and B16/APJ-KO tumors, we postulated that blood supply other than through tumor vessels is required. Therefore, we speculated that vascular mimicry (VM) may be induced in B16/APJ-KO tumors and indeed we detected abundant periodic acid Schiff (PAS)-positive, but CD31-negative, vascular-like structures in tumors generated by B16/APJ-KO cells compared to those of B16/WT cells (Figures 4A, B).

It is widely accepted that a change in cell shape is involved in vascular mimicry by tumor cells [19]. We found that APJ-KO B16 cells showed a more spindle-like shape compared to WT cells (Figure 4C). Several studies suggested that VM by B16 cells requires cancer cell migration. We therefore compared the migration of B16/WT and B16/APJ-KO cell lines using transwell assays. We found that B16/APJ-KO (#1 and 2) cells showed more transmigration than B16/WT cells (Figures 4D, E). Moreover, an *in vitro* tube formation assay that mimics vascular formation indicated that the lack of APJ more effectively generated tube-like structures in B16 cells (Figures 4F, G).

### Lack of APJ in B16 cells induces high Zeb1 expression by not correlating with TGF-β/smad signaling pathways

**FIGURE 5:**
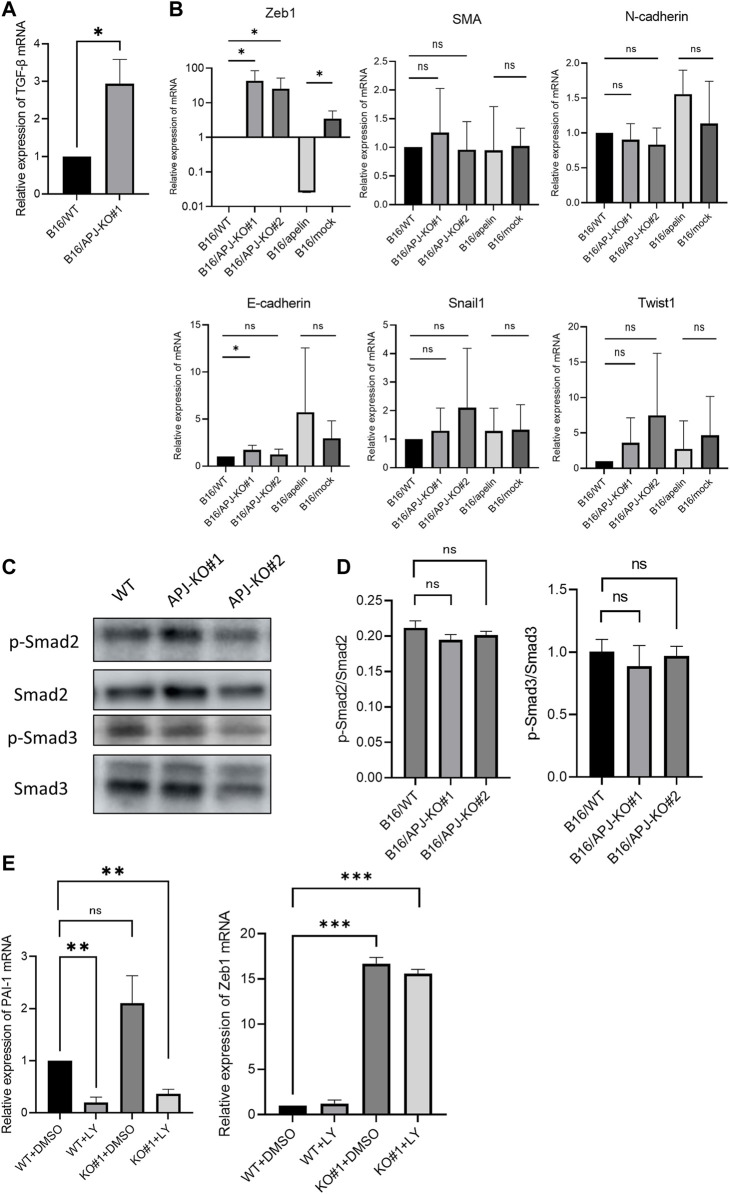
APJ knock out in B16 cells shows persistently high Zeb1 expression uncorrelated with TGF-β/smad signaling. **(A)** Quantitative evaluation of transforming growth factor (TGF)-β mRNA expression in B16/WT and B16/APJ-KO#1 cells (*n* = 3). **(B)** Quantitative evaluation of epithelial–mesenchymal (EMT) markers (Zeb1, smooth muscle actin [SMA], N-cadherin, E-cadherin, Snail, Twist1) mRNA expression in B16/wild-type (WT), B16/APJ-knockout (KO) (#1, 2), and B16/apelin and B16/mock-transfected cells (*n* = 4). **(C)** Western blot analysis of phosphorylated Smad2 (p-Smad), Smad2, phosphorylated Smad3 (p-Smad3), and Smad3 protein levels in cell lysates of B16/WT (WT) and B16/APJ-KO#1,2 (APJ-KO#1, 2) cells. **(D)** Quantitative evaluation of the p-Smad2/Smad protein ratio and p-Smad3/Smad3 protein ratio in B16/WT and B16/APJ-KO cells (*n* = 4). **(E)** Quantitative evaluation of plasminogen activator inhibitor-1 (PAI-1) and Zeb1 mRNA expression in WT and APJ-KO cells after treatment with dimethyl sulfoxide (DMSO) (control) or LY364947 (*n* = 3). Data are expressed as mean ± SD, *p < 0.05, **p < 0.01. ***p < 0.001. ns, not significant.

Thus far, we found that a lack of APJ in B16 cells meant that these showed a spindle-like phenotype with high migrating potency. We hypothesized that this phenotypic change may be induced by mechanisms involved in epithelial–mesenchymal Transition (EMT). Signaling affected by transforming growth factor (TGF)-β is a key factor for EMT. Therefore, we examined the expression of TGF-β and transcription factors associated with EMT. We found that TGF-β mRNA was expressed in B16/APJ-KO cells at a higher level than in B16/WT cells (Figure 5A). Correlating with this expression, expression of the mesenchymal maker, Zeb1, was also higher in APJ-KO melanoma and B16/apelin cells than in B16/WT cells (Figure 5B). By contrast, when smooth muscle actin (SMA) mRNA expression was observed, a slightly higher level was detected in B16/apelin compared to B16/mock-transfected cells; however, an increase in SMA mRNA was not detected in B16/APJ-KO cells. Since the expression of another EMT-related molecule was not altered in B16/apelin or B16/APJ-KO cells, as far as we could determine, this phenotypic change by APJ or apelin may have been induced by partial EMT. Recently, it has been reported that partial EMT status prior to the acquisition of full mesenchymal phenotype provides maximal stemness, tumor initiation capacity, and the ability to adapt to environmental changes [20].

Next, we investigated the relationship of TGF-β/Smad signaling with Zeb1 expression. At first, phosphorylated Smad2 and Smad3 (pSmad2 and pSmad3) proteins were measured by western blotting in B16 cells. However, we found that the ratio of pSmad2/Smad2 and pSmad3/Smad3 proteins were not altered by the absence of APJ (Figures 5C, D). Next, we further observed the relationship between Zeb1 and TGF-β/Smad signaling by using an inhibitor of TGF-β/Smad signaling pathways. We showed that an inhibitor of the TGF-β1 receptor (LY364947) affected B16/WT and B16/APJ-KO cells since the mRNA level of plasminogen activator inhibitor-1, which is an end-product of the Smad signaling pathway, was inhibited. In this setting, however, Zeb1 expression was not suppressed by LY364947 in the B16/APJ-KO group (Figure 5E). Therefore, we concluded that APJ negatively regulated Zeb1 expression in B16 cells and therefore attenuation of APJ in B16 cells promoted Zeb1 expression to induce EMT; however, this APJ signaling is independent of TGF-β/Smad signaling pathways.

## Discussion

In subcutaneous inoculation of tumor cell mouse models, we previously reported that overexpression of apelin in colon 26 tumor cells significantly inhibited tumor growth by promoting penetration of natural killer T cells into the tumor. Additionally, [Pyr1] apelin-13 infusion induced CD8^+^  T-cell infiltration into tumors, resulting in inhibition of tumor growth through C-C Motif Chemokine Ligand 8 expression [13]. In contrast to our previous findings, several studies have shown that the apelin/APJ axis plays a role in the development of tumors. In many types of tumors, it has been shown that apelin and APJ expression levels are significantly increased. For example, in the lung adenocarcinoma cell line A549, apelin increases cyclin D1 expression and induces cell proliferation through enhancement of extracellular signal–regulated kinase (ERK)1/2 phosphorylation [21]. In the case of colon cancer, apelin and APJ expression is elevated in tissues of colon adenocarcinoma and the human colon carcinoma cell line LS180 [22]. Apelin induces the proliferation of LS180 cells *via* upregulation of Notch3 resulting in the activation of JAG1/Notch3 signaling. Also, in the human oral cancer cell line, HSC-3, apelin promotes cell proliferation through phosphorylation of ERK1/2 [23]. Thus, the effect of apelin on the cancer cell itself is thought to be involved in tumor growth. In the present study, to clarify the effect of apelin in cancer cells, we used APJ-KO mice as a host in subcutaneous tumor cell inoculation models. Based on our present studies, we showed that apelin has a role in tumor progression, affecting tumor cells directly. Taken together with our previous reports, it was elucidated that apelin has dual roles in tumor growth, i.e., cancer cell proliferation directly affecting APJ in cancer cells and a cancer-suppressive effect by enhancing tumor immunity through the tumor vasculature.

To date, reports that highlight the relationship between APJ and VM are non-existent. Vascular mimicry, in which cancer cells themselves display vascular-like structures to maintain blood flow in the absence of blood vessels covered with vascular ECs, supports tumor growth. The mechanism of VM has not been well elucidated compared with tumor blood vessels regulated by angiogenesis mainly induced by VEGF. The process of VM is induced by signaling pathways associated with hypoxia, cancer stemness, and angiogenesis [24]. Of these pathways, hypoxia is associated with VM in melanoma [25]. Hypoxia induces VM formation by upregulating VE-cadherin expression. Hypoxia-inducible factor 1, a key effector of this pathway, increases the stemness and differentiation potential of highly plastic cells in highly malignant tumor cell populations (especially cancer stem cells) in the tumor microenvironment. Such tumor cells transform into more mobile cells through hypoxia-induced phenotype change. Then, as the cells expand and contract, the expression of related transcription factors, such as Twist and Snail, in cells increases, the expression of E-cadherin decreases, and the expression of angiogenesis-related molecules such as fibronectin increases [26]. These cancer stem cells subsequently show characteristics of endothelial-like cells [27].

Like hypoxia, VEGF is an important factor in angiogenesis. Vascular endothelial growth factor expression in melanomas promotes VM development through activation of the PI3K/AKT pathway in the tumor microenvironment [28]. Phosphoinositide 3-kinase elevates the level of matrix metalloproteinase-14 (MMP-14) in aggressive cells; MMP-14 in turn activates MMP-2, which in turn activates the secretion of γ2′ and γ2x pro-migratory fragments leading to VM in melanomas [29]. It restructures the extracellular matrix, facilitates tumor cell migration and conversion to a malignant phenotype, and provides the stretch space and environment for VM network formation [30].

Several VM inhibitors to suppress these pathways have been identified in previous studies. For example, molecules suppressing VEGF/VEGF receptor pathways, such as thalidomide, efficiently inhibit VM [31,32]. However, the other angiogenesis-related ligand/receptor pair associated with VM has not been well documented. Here, we revealed that APJ expression in cancer cells negatively correlated with the formation of VM. In brief, the lack of APJ benefits B16 cells in promoting VM.

Several studies have reported that increased expression of Zeb1 in melanoma is involved in phenotype change into an invasive one [33]. In our study, we also detected that a high migration state in APJ-KO B16 cells is accompanied by high Zeb1 expression. Epithelial–mesenchymal-like phenotypic change is also associated with VM formation [26, 34]. Interestingly, in glioblastoma, a representative tumor that shows VM, a reduction in apelin expression led to accelerated glioblastoma cell invasion [35]. Taken together with our present data, more tumor cell types are likely to exist that show an aggressive migration phenotype due to a lack of apelin/APJ expression.

In summary, depending on tumor cell types, apelin may act as a promotor of tumor proliferation. However, apelin is a regulator of mature blood vessel formation in both physiological and pathological settings. Based on the analysis shown in this report, we interpret that specific regulation of APJ expression in tumor cells is required to control tumor growth by affecting cancer cells themselves. The regulation of APJ expression in cancer cells is currently unknown. We need to analyze the regulation of APJ expression in tumor cells, including epigenetic regulation. In the present study, we did not specifically elucidate how an EMT-like phenotype or VM was induced by a lack of APJ in B16 melanoma cells, although Zeb1 expression was apparently induced. Further analysis to determine molecular insights of how Zeb1 is negatively regulated by APJ in cancer is required.

## Data Availability

The original contributions presented in the study are included in the article/Supplementary Material, further inquiries can be directed to the corresponding author.
