# Supplementary material for: Cancer apelin receptor suppresses vascular mimicry in malignant melanoma
Source: Pathol Oncol Res. 2023 Jan 27;29:1610867. doi: 10.3389/pore.2023.1610867 (PMC9912982; doi:10.3389/pore.2023.1610867)
Supplement: Supplementary file 1 [file Table1.DOCX]

**Supplemental Table 1 Oligonucleotide sequences for PCR**

|  | Gene | Primer sequence |
| --- | --- | --- |
| Real-time PCR | *Mouse Apelin* | 5’-GTGCCCTCCCGGTGCCGGTCTCT-3’  5’-GAGACCACGCCATTAGAGGAACT-3’ |
|  | *Mouse APJ* | 5’-CCACTGTGGGCCACTTATACC-3’  5’-CAGCCTTAGCCGAGCATTG-3’ |
|  | *Mouse ZEB1* | 5’-GGAAGAGAGCAAAGACATGTGA-3’  5’-AAACCGTTTCTTGCAGTTCG-3’ |
|  | *Mouse SMA* | 5’-CTTCGCTGGTGATGATGCTC-3’  5’-GCGTCAGGATCCCTCTCTTG-3’ |
|  | *Mouse E-cadherin* | 5’-TGACCAGCAGTTCGTTGTTG-3’  5’-CCTCAAAGGGTTCCTCGTTC-3’ |
|  | *Mouse N-cadherin* | 5’-CGCAGTCTTACCGAAGGATG-3’  5’-CTGGCTCGCTGCTTTCATAC-3’ |
|  | *Mouse Snail* | 5’-CAAGGAGTACCTCAGCCTGG -3’  5’-GTCAGCAAAAGCACGGTTG-3’ |
|  | *Mouse Twist1* | 5’-CTGCCCTCGGACAAGCTGAG -3’  5’-CTAGTGGGACGCGGACATGG -3’ |
|  | *Mouse PAI-1* | 5’-CCACAAAGGTCTCATGGACCAT -3’  5’-TGAAAGTGTTGTGCCCTCCAC -3’ |
|  | *Mouse GAPDH* | 5’-CTAGCTGTGCCTGTCATGG-3’  5’-GGCTGACTTCTCGGCACTAC-3’ |
